# Insights into the Pathology of the α_3_ Na^+^/K^+^-ATPase Ion Pump in Neurological Disorders; Lessons from Animal Models

**DOI:** 10.3389/fphys.2016.00209

**Published:** 2016-06-14

**Authors:** Thomas H. Holm, Karin Lykke-Hartmann

**Affiliations:** ^1^Department of Biomedicine, Aarhus UniversityAarhus, Denmark; ^2^Department of Molecular Biology and Genetics, Centre for Membrane Pumps in Cells and Disease-PUMPKIN, Danish National Research Foundation, Aarhus UniversityAarhus, Denmark; ^3^Aarhus Institute of Advanced Studies, Aarhus UniversityAarhus, Denmark

**Keywords:** α_3_ sodium ion pump, neurons, rapid onset dystonia parkinsonism (RDP), alternating hemiplegia of childhood (AHC) and cerebellar ataxia, areflexia, pes cavus, optic atrophy, and sensorineural hearing loss (CAPOS), mouse models

## Abstract

The transmembrane Na^+^-/K^+^ ATPase is located at the plasma membrane of all mammalian cells. The Na^+^-/K^+^ ATPase utilizes energy from ATP hydrolysis to extrude three Na^+^ cations and import two K^+^ cations into the cell. The minimum constellation for an active Na^+^-/K^+^ ATPase is one alpha (α) and one beta (β) subunit. Mammals express four α isoforms (α_1−4_), encoded by the *ATP1A1-4* genes, respectively. The α_1_ isoform is ubiquitously expressed in the adult central nervous system (CNS) whereas α_2_ primarily is expressed in astrocytes and α_3_ in neurons. Na^+^ and K^+^ are the principal ions involved in action potential propagation during neuronal depolarization. The α_1_ and α_3_ Na^+^-/K^+^ ATPases are therefore prime candidates for restoring neuronal membrane potential after depolarization and for maintaining neuronal excitability. The α_3_ isoform has approximately four-fold lower Na^+^ affinity compared to α_1_ and is specifically required for rapid restoration of large transient increases in [Na^+^]_i_. Conditions associated with α_3_ deficiency are therefore likely aggravated by suprathreshold neuronal activity. The α_3_ isoform been suggested to support re-uptake of neurotransmitters. These processes are required for normal brain activity, and in fact autosomal dominant *de novo* mutations in *ATP1A3* encoding the α_3_ isoform has been found to cause the three neurological diseases Rapid Onset Dystonia Parkinsonism (RDP), Alternating Hemiplegia of Childhood (AHC), and Cerebellar ataxia, areflexia, pes cavus, optic atrophy, and sensorineural hearing loss (CAPOS). All three diseases cause acute onset of neurological symptoms, but the predominant neurological manifestations differ with particularly early onset of hemiplegic/dystonic episodes and mental decline in AHC, ataxic encephalopathy and impairment of vision and hearing in CAPOS syndrome and late onset of dystonia/parkinsonism in RDP. Several mouse models have been generated to study the *in vivo* consequences of *Atp1a3* modulation. The different mice show varying degrees of hyperactivity, gait problems, and learning disability as well as stress-induced seizures. With the advent of several *Atp1a3*-gene or chemically modified animal models that closely phenocopy many aspects of the human disorders, we will be able to reach a much better understanding of the etiology of RDP, AHC, and CAPOS syndrome.

## The Na^+^/K^+^ ATPases: expression and function

The Na^+^/K^+^ ATPases are transmembrane ion-pumps located at the plasma membrane of all mammalian cells. With each pump cycle, the Na^+^/K^+^ ATPases utilize the energy from hydrolysis of one adenosine triphosphate (ATP) to extrude three Na^+^ ions and import two K^+^ ions into the cell. The minimum constellation of an active pump consists of an alpha (α) and a beta (β) subunit (Kaplan, 2002; Bublitz et al., 2011; Palmgren and Nissen, 2011). The α subunit is responsible for the catalytic and pharmacological properties (Blanco et al., 1994) whereas the β and optional γ subunits may have regulatory functions (Jaisser et al., 1994; Béguin et al., 1997; Hilbers et al., 2016).

Mammals express four Na^+^/K^+^ ATPase α isoforms (α_1−4_) of which α_1−3_ are expressed in the CNS. While α_1_ is expressed ubiquitously and considered to maintain housekeeping cellular functions, the α_2_ isoform is expressed primarily in astrocytes and developing neurons and α_3_ isoform is restricted to neurons (McGrail et al., 1991; Bøttger et al., 2011). Thus, the cell type-specific distribution of the α isoforms suggests that each isoform has a particular function. Several suggestions have been made to elaborate on the specific role of the α_3_ isoform in neurons (Reviewed in Dobretsov and Stimers, 2005). Although the α_3_ isoform is expressed in many CNS neurons, several neuronal subsets lack the expression of this isoform (Hieber et al., 1991; McGrail et al., 1991; Bøttger et al., 2011). The ongoing hypothesis is thus that while the ubiquitously expressed α_1_ isoform maintains neuronal housekeeping functions, the α_3_ isoform serves as a reserve pump that only becomes activated when the intracellular Na^+^ concentration [Na^+^]_i_ is high, e.g., after repeated action potentials (Brodsky and Guidotti, 1990; Jewell and Lingrel, 1991; Munzer et al., 1994; Blanco et al., 1995; Zahler et al., 1997; Monteith and Blaustein, 1998; Crambert et al., 2000), supported by the fact that the kinetics between the different isoform favors this scenario (Reviewed in Dobretsov and Stimers, 2005).

## Neuronal activity and α_3_

In the CNS, the Na^+^ and K^+^ gradients across the plasma membrane are essential for regulating neuronal excitability, and for multiple cellular functions, including cell volume regulation and Na^+^-coupled secondary transport. The distinguishing feature of α_3_ Na^+^/K^+^-ATPases is their several-fold lower affinity for activation by cytoplasmic Na^+^ compared to that of α_1_ Na^+^/K^+^-ATPases (Crambert et al., 2000). In rapidly firing neurons, therefore, when action potentials increase the intracellular Na^+^ concentration, [Na^+^]_i_, beyond saturating levels of the “housekeeping” α_1_ Na^+^/K^+^-ATPases, activation of α_3_ Na^+^/K^+^-ATPases continues to increase as [Na^+^]_i_ rise. The α_3_ isoform thus protects neurons against catastrophic elevation of [Na^+^]_i_ (Dobretsov and Stimers, 2005), and also of [Ca^2+^]_i_ (because Na^+^/Ca^2+^ exchange is weakened) and general loss of the Na^+^ electrochemical gradient. As the α_3_ isoform is detected in several basal ganglia neuronal subsets (McGrail et al., 1991; Bøttger et al., 2011), reduced α_3_ activity may therefore interfere with reuptake of neurotransmitters such as glutamate, γ-aminobutyric acid (GABA) and dopamine (Kristensen et al., 2011), in those neurons.

The neurotransmitter transporters (NTTs) use ion gradients for the active transport, generally by co-transport of Na^+^ and Cl^−^. As an example, the glycine transporters are functionally coupled to the Na^+^ electrochemical gradient, which is actively generated and maintained by the Na^+^/K^+^-ATPases. The glycine transporter, GlyT2 cotransports three Na^+^ and one Cl^−^ for every glycine (López-Corcuera et al., 1998), generating large rises in [Na^+^]_i_ that must be efficiently reduced by the Na^+^/K^+^-ATPase to preserve ion homeostasis, which is absolutely necessary for synaptic transmission and neuronal excitability. The α_3_ co-localize and interacts with GlyT at the synapse in spinal cord neurons (de Juan-Sanz et al., 2013). As GlyT belongs to the solute carrier 6 (SLC6) family of highly homologous NTTs, it is possible that the transporter for dopamine, norepinephrine, serotonin, and GABA can affected by Na^+^/K^+^-ATPase activity loss (Kristensen et al., 2011). It is well-documented that dopamine increases the activity of the Na^+^/K^+^-ATPase in an organ-specific manner (Reviewed in Zhang et al., 2013). Moreover, evidence of direct interaction between Na^+^/K^+^-ATPase and dopamine receptors has also been reported (Hazelwood et al., 2008). Dopamine modulates the Na^+^/K^+^-ATPase by affecting both dopamine and other hormones. It has been shown that the D2 receptor stimulates striatal Na^+^/K^+^-ATPase activity after a short-term morphine treatment, in contrast to a long-term morphine treatment that inhibited striatal Na^+^/K^+^-ATPase activity (Wu et al., 2007).

Failure of the Na^+^/K^+^-ATPase to maintain Na^+^ and K^+^ gradients will cause a decrease in Na^+^ and K^+^ currents (through voltage-dependent channels). This will lead to a decrease in the membrane potential, action potential and most likely a loss of neuronal excitability. This will further affect sodium-coupled co- and counter-transport. Moreover, it can also affect Na^+^/H^+^ and Na^+^/Ca^2+^ exchange, essential for cellular functions, and could lead to increased intracellular Ca^2+^ and acidification. Not surprisingly, mutations in the *ATP1A3* gene have been associated with neurological disorders.

## *ATP1A3*-related diseases

Mutations in the *ATP1A3* gene are associated with three related rare neurological disorders, Rapid-onset Dystonia-Parkinsonism (RDP) (de Carvalho Aguiar et al., 2004), Alternating Hemiplegia of Childhood (AHC) (Heinzen et al., 2012; Rosewich et al., 2012), and recently, Cerebellar ataxia, Areflexia, Pes cavus, Optic atrophy, and Sensorineural hearing loss (CAPOS) syndrome (Demos et al., 2014).

Presently 12 *ATP1A3* missense mutations have been associated with RDP (Heinzen et al., 2014). Classical RDP patients typically develop stress-induced permanent dystonia and Parkinsonism in late adolescence or early adulthood. Other 59 different *ATP1A3 de novo* missense mutations are associated with AHC (Heinzen et al., 2014; Rosewich et al., 2014; Sasaki et al., 2014; Ulate-Campos et al., 2014; Yang et al., 2014; Panagiotakaki et al., 2015; Viollet et al., 2015). AHC is characterized by onset of hemiplegic/quadriplegic episodes within 18 months of birth. Other, more variable neurological abnormalities of AHC include choreathosis, ataxia, developmental delays, seizures, and high incidence of neuropsychiatric disorders (Demos et al., 2014).

Recently, the mutations causing RDP and AHC were mapped according to their amino acid position in the α_3_ isoform showing their location and the number of patients identified that harbors these mutations (Heinzen et al., 2014).

So far, only a single missense mutation in *ATP1A3* is associated with CAPOS syndrome (Demos et al., 2014; Heimer et al., 2015). CAPOS patients show onset of symptoms at the age of 6 months to 5 years. CAPOS syndrome is characterized by episodes of ataxic encephalopathy, weakness, and loss of hearing and sight (Brashear et al., 2014).

Interestingly, a recent report identified a G316S mutation in the α_3_ isoform associated with Adult Rapid-onset Ataxia (Sweadner et al., 2016). The clinical examination noted most RDP symptoms except dystonia.

Thus, it appears that the *ATP1A3*-related disorders arise from autosomal dominant mutations with variable penetrance (Demos et al., 2014). Affected patients typically present in the context of an acute onset of paroxysmal, episodic neurological symptoms that may include hemiplegia, dystonia, ataxia, or seizures. Some symptoms may persist after resolution, such as neurodevelopmental delays, attention deficits, hyperactivity, trunk instability, dystonia, or ataxia (Mikati et al., 2000; Shafer et al., 2005; Panagiotakaki et al., 2010; Heinzen et al., 2012, 2014; Sweney et al., 2015). Although the *ATP1A3*-related neurological disorders are considered clinically distinct, the phenotypic spectrum of each disease continues to expand (Heinzen et al., 2014; Rosewich et al., 2014; Sweney et al., 2015). In support, there have recently been identified patients with intermediate, non-classical symptoms (Sasaki et al., 2013; Termsarasab et al., 2015).

## Genotype-phenotype—affect protein function—cause symptoms

A recent case study of 35 AHC patients showed relatively mild symptoms in patients carrying the D801N mutation, whereas the E815K mutation was associated with far more severe symptoms (Sasaki et al., 2014). In fact, the same *ATP1A3* mutation may result in quite different time of onset and disease progression (Dobyns et al., 1993; Oblak et al., 2014), emphasizing that other factors, such as genetic background and epigenetic regulation play a large role in disease penetrance.

Recent functional studies suggest that most RDP mutations do not reach the cell surface (Heinzen et al., 2012). In contrast, the majority of AHC mutant proteins exert dominant negative effects on the wild type protein at the cell surface (Clapcote et al., 2009; Li et al., 2015), thus explaining the more severe phenotypes associated with AHC. The majority of AHC mutations (>70%) are located within the transmembrane helices of the Na^+^/K^+^-ATPase protein (Heinzen et al., 2012). Homology modeling of the two most common mutations, D801N and E815K suggested different mutational consequences: In the D801N mutation, a positive dipole was formed, which through electrostatic repulsion directly affected passage of K^+^ ions (Kirshenbaum et al., 2013). In contrast, the E815K mutation prevented inward H^+^ currents through the Na^+^/K^+^-ATPase. Intracellular H^+^ currents through the Na^+^/K^+^-ATPase were recently identified (Vedovato and Gadsby, 2014) and are known regulators of neuronal excitability (Takahashi and Copenhagen, 1996), thus suggesting a correlation between severity and loss of H^+^ transport. The significance of this discovery however remains to be determined *in vivo*.

## AT*P1A3*-modified mouse models

Four genetically modified mouse models targeting the *Atp1a3* gene has been reported, and have been extensively used to study *in vivo* functions of the α_3_ isoform (Table 1). A detailed comparison of mouse models looking at clinical features in AHC and *Atp1a3* mouse models, as well as behavioral testing has been reported (Hunanyan et al., 2015).

**Table 1:** ***Atp1a3* gene modified animals**.

Mouse model^*a^	***Atp1a3* genetic alteration**	**Major behavioral observations**	**References**
*Atp1a3*^tm1Ling∕+^ ($\alpha_{3}^{+∕\text{KOI}4}$)	Deletion targeting intron 4	Increased locomotor activity	Moseley et al., 2007; DeAndrade et al., 2011; Kirshenbaum et al., 2011b
		Increased methamphetamine response	
		Intact grip strength	
		Spatial learning impairment Stress-induced symptoms Motor deficits Sensory system defects Impaired novel object recognition Despair Anhedonia Increased anxiety Reduced learning and memory Reduced sociability	
$\alpha_{3}^{+∕\Delta \text{E}2-6}$	Deletion targeting exon 2–6	Increased locomotor activity	Ikeda et al., 2013; Sugimoto et al., 2014
		Increased dystonic response to intracerebellar kainate injections	
		Enhanced inhibitory neurotransmission	
		Intact grip strength	
		Enhanced motor balance	
		Stress-induced motor deficits	
*Myshkin* ($\alpha_{3}^{+∕\text{I}810\text{N}}$)	Single nucleotide substitution causing a single amino acid substitution (I810N)	Increased locomotor activity	Clapcote et al., 2009; Kirshenbaum et al., 2011a, 2012, 2013
		Increased methamphetamine response	
		Spontaneous epileptic seizures	
		Neuronal hyperexcitability	
		Reduced learning and memory	
		Ataxia	
		Mania-like behavior	
Mashl^+∕−^ ($\alpha_{3}^{+∕\text{D}801\text{N}}$) (Mashlool)	Single nucleotide substitution causing a single amino acid substitution (D801N)	Increased locomotor activity	Hunanyan et al., 2015
		Spontaneous epileptic seizures	
		Neuronal hyperexcitability	
		Reduced learning and memory	
		Ataxia	
		Dystonia	
		Hemiplegia	

*a *Two Atp1a3 knock-out (KO) and two knock-in (KI) mice have been described. For all four models, only heterozygous animals are viable after birth*.

The *Atp1a3*^tm1Ling∕+^ was produced by introducing a single base pair mutation in intron 4 ($\alpha_{3}^{+∕\text{KOI}4}$), causing an aberrant splicing of *Atp1a3*, effectively knocking out the allele (Moseley et al., 2007). The $\alpha_{3}^{+∕\Delta \text{E}2-6}$ was generated by an eGFP-*Atp1a3* gene replacement strategy, knocking out one *Atp1a3* allele ($\alpha_{3}^{+∕\Delta \text{E}2-6}$) (Ikeda et al., 2013). The *Myshkin* mouse ($\alpha_{3}^{+∕\text{I}810\text{N}}$) (Clapcote et al., 2009) expressed the I810N AHC mutation (Panagiotakaki et al., 2015). The Mashlool mouse ($\alpha_{3}^{+∕\text{D}801\text{N}}$, Mashl^+∕−^) (Hunanyan et al., 2015) was generated to study the effects of the most common mutation in AHC, D801N (Heinzen et al., 2012).

The cardiotonic steroids (CTS) constitute a group of organic compounds that show high binding affinity toward Na^+^/K^+^-ATPases and inhibit the catalytic activity by stabilizing the enzyme's phosphorylated E2-P state (Yatime et al., 2011; Laursen et al., 2013). CTS such as ouabain have been used for more than 50 years to study the function of Na^+^/K^+^-ATPases *in vitro* and in acute animal models. An ouabain-perfused mouse model presumably targeting the α_3_ isoform in cerebellum and basal ganglia (Calderon et al., 2011) supplements the findings summarized in Table 1.

## Basic characterization

Hippocampal lysates showed a significant reduction in α_3_ protein expression in the $\alpha_{3}^{+∕\text{KOI}4}$ mice and a compensatory upregulation of α_1_ expression but not of α_2_ (Moseley et al., 2007). In contrast, whole brain lysates from the *Myshkin* mice (Myk/+ and Myk/Myk) showed no changes in protein expression levels of the α_1_, α_2_, and α_3_ isoforms relative to wild type, suggesting that the *Myshkin* mutation is a functional null allele of the *Atp1a3* gene, which encodes a normally expressed, but inactive enzyme, as revealed by reduced total ATPase activity (Clapcote et al., 2009). In support of this, the authors observed normal plasma membrane localization of the *Myskhin* α_3_ isoform expressed in COS cells with only small amounts retained in the ER (Clapcote et al., 2009).

AHC patients tend to be smaller and weigh less—presumably due to eating difficulties (Neville and Ninan, 2007; Panagiotakaki et al., 2010). Although abilities to chew and swallow have not been addressed for any of the mouse models, it is interesting that the *Myshkin* and male Mashl^+∕−^ mice were reported to have smaller body sizes (Clapcote et al., 2009; Hunanyan et al., 2015). *Myshkin* mice crossed with mice expressing a bacterial artificial chromosome expressing wild type *Atp1a3* (*Atp1a3* BAC) regained approximately 80% of wild type total Na^+^/K^+^-ATPase activity and showed normal body size, thus supporting the theory that Na^+^/K^+^-ATPase activity loss correlates with symptoms (Clapcote et al., 2009).

## Effects on motor function, balance, and coordination

The motor dysfunction in RDP and AHC i.e., ataxia, dystonia, unsteady gait, and tip-toing are reflected in motor function, balance problems, and gait disturbances (Brashear et al., 1997; Linazasoro et al., 2002; Svetel et al., 2010).

Combined, the *Atp1a3*^tm1Ling∕+^ and $\alpha_{3}^{+∕\Delta \text{E}2-6}$ mouse models displayed mild motor symptoms, whereas the *Myshkin* and the Mashl^+∕−^ mice recapitulated a much broader spectrum of *ATP1A3*-disease related symptoms. Motor tests showed these defects were caused by dyscoordination rather than lack of muscle strength as grip strength was intact (Kirshenbaum et al., 2013; Hunanyan et al., 2015), and thus corresponds to reports of AHC patients.

Adult *Atp1a3*^tm1Ling∕+^ and $\alpha_{3}^{+∕\Delta \text{E}2-6}$ mice displayed normal motor function when tested on the balance beam and accelerated rotarod (DeAndrade et al., 2011; Sugimoto et al., 2014). However, restraint caused a stress-induced deterioration of motor performance of mice on the balance beam and rotarod and a significant drop in ATPase activity from 85 to 67% (DeAndrade et al., 2011).

Similarly, restraint stress significantly reduced the hanging times of the $\alpha_{3}^{+∕\Delta \text{E}2-6}$ mice (Sugimoto et al., 2014). In contrast to *Atp1a3*^tm1Ling∕+^ and $\alpha_{3}^{+∕\Delta \text{E}2-6}$ mice, distinct motor symptoms were apparent in naïve *Myshkin* and the Mashl^+∕−^ mice: Both strains showed ataxia and tremor on the balance beam but only the *Myshkin* mice performed poorly on the accelerated rotarod (Kirshenbaum et al., 2013; Hunanyan et al., 2015).

Both *Myshkin* and the Mashl^+∕−^ mouse strains showed abnormal stride length (Kirshenbaum et al., 2013; Hunanyan et al., 2015). A similar phenotype was induced by restraint stress in the $\alpha_{3}^{+∕\Delta \text{E}2-6}$mice (Sugimoto et al., 2014).

## The cerebellum-basal ganglia circuitry

### Dystonia, ataxia, and parkinsonism

Dystonia, ataxia, and Parkinsonism are core symptoms of RDP. A recent study showed that dystonia and ataxia could be reproduced by partially blocking the Na^+^/K^+^-ATPase in the cerebellum of mice using ouabain whereas disruption of the basal ganglia circuit caused Parkinsonism i.e., rigidity, akinesia, tremor, and hunched posture (Calderon et al., 2011). The symptoms induced by ouabain perfusion were shown to develop over several days and to be highly dependent on the ouabain concentration used. Interestingly, mice perfused with low concentrations of ouabain into the cerebellum and basal ganglia developed symptoms corresponding to high concentration of ouabain when subjected to stress. Thus, reflecting the stress-sensitive nature of *ATP1A3*-related diseases.

Cerebellar dysfunction has been observed in several rodent dystonia strains (Lorden et al., 1992; LeDoux and Lorden, 1998; Richter et al., 1998; Campbell et al., 1999; Fremont et al., 2014) and in patients suffering from AHC (Saito et al., 1998; Yamashita et al., 2005; Sasaki et al., 2009) and RDP (Oblak et al., 2014; Liu et al., 2015).

The $\alpha_{3}^{+∕\Delta \text{E}2-6}$ mice showed prolonged dystonic periods after intracerebellar kainate injections (Ikeda et al., 2013). Despite reduced motor performance of both naïve *Myshkin* and the Mashl^+∕−^ mice, only the Mash^+∕−^ mice were reported to develop stress-induced generalized dystonia (Hunanyan et al., 2015). Mild dystonia is common among AHC patients carrying the Mashl^+∕−^ equivalent D801N mutation (Panagiotakaki et al., 2015), whereas only one of two AHC patients known to carry the *Myskhin*, I810N mutation, showed dystonia (Yang et al., 2014; Panagiotakaki et al., 2015). Presently, it is therefore not possible to determine if the differences observed can be attributed specifically to the *ATP1A3* mutation or to genetic background.

The deep cerebellar nuclei (DCN) integrate inhibitory signals from GABAergic Purkinje neurons and excitatory glutamatergic mossy fibers and climbing fiber pathways and constitute the majority output fibers from the cerebellum. The DCN connects to premotor and motor nuclei in the basal ganglia (Faull, 1978; Faull and Carman, 1978). Abnormal output from the DCN will have profound effects on motor function. The Purkinje neurons are the main output to the DCN and play a central role in DCN signal integration and overall cerebellar function.

Purkinje neurons are characterized by a high Na^+^ channel density and short spike duration during which large amounts of Na^+^ ions enter the neurons, emphasizing the requirement for functional a Na^+^/K^+^-ATPase (Llinás and Sugimori, 1980; Raman and Bean, 1997; Carter and Bean, 2009). Cerebellar Purkinje neurons only express the Na^+^/K^+^-ATPase α_3_ isoform (Peng et al., 1997) and are therefore particularly sensitive to *ATP1A3* mutations. Furthermore, in developing $\alpha_{3}^{+∕\Delta \text{E}2-6}$mice, reduced Na^+^/K^+^-ATPase α_3_ expression caused a build-up of [Na^+^]_i_ and [Ca^2+^]_i_ in the terminals of molecular-layer interneurons resulting in enhanced inhibition of Purkinje neurons (Ikeda et al., 2013). This observation is potentially very interesting, as it offers a possible mechanism to explain how Na^+^/K^+^-ATPase perturbations may affect neuronal plasticity and motor learning.

Although further experiments are required to determine the extent of this observation, it is interesting, that cortical neurons isolated from the *Myshkin* mice showed increased [Ca^2+^]_i_ as well (Kirshenbaum et al., 2011a). This observation could imply that other neuronal populations may be affected. Indeed, Na^+^/K^+^-ATPase α_3_ showed co-localization with markers for several populations of GABAergic neurons in the rodent and human brain (Bøttger et al., 2011; Ikeda et al., 2013; Paciorkowski et al., 2015), suggesting these populations to be sensitive to *ATP1A3* perturbations as well. Supporting GABA dysfunction, hippocampal neurons from the *Myshkin* and Mashl^+∕−^ mice showed increased excitability in response to high frequency stimulation (Clapcote et al., 2009; Hunanyan et al., 2015).

## Hemiplegia

Hemiplegia is specific to AHC (Sweney et al., 2015). The mechanism driving this symptom is poorly understood. Single-photon emission computed tomography (SPECT) scans of AHC patients showed malperfusion leading up to or during events in several cases (Kanazawa et al., 1991; Zupanc et al., 1991; Aminian et al., 1993; Wong et al., 1993). However, there have also been examples, where this phenotype was absent (Sweney et al., 2009; Sasaki et al., 2011). Supporting a vascular phenotype, the vasodilator, flunarizine is currently among the most widely used prophylaxis of hemiplegia in AHC (Casaer and Azou, 1984; Bourgeois et al., 1993; Mikati et al., 2000). So far, there has been no investigation of this mechanism in the current animal models. Of the four mouse models, only the Mashl^+∕−^ mice were reported to develop stress-induced episodes of hemiplegia and quadriplegia (Hunanyan et al., 2015).

## Neuropsychiatric symptoms—mania

Previous studies have shown intracerebroventricular (ICV) injections of ouabain in high concentrations to be CNS stimulatory and convulsive (Doggett and Spencer, 1971; Davidson et al., 1978; Corazzi et al., 1985; Haglund and Schwartzkroin, 1990; Lees et al., 1990; Yu, 2003). In rats, this approach was used to induce mania (El-Mallakh et al., 2003; Riegel et al., 2009). Episodes mimicking manic periods of bipolar mood disorder are common among AHC patients. Especially the manic episodes are particularly detrimental, as the children are at high risk of injury (Personal communications, see Acknowledgement).

There is a strong link in literature between Na^+^/K^+^-ATPase dysfunction and bipolar mood disorder (el-Mallakh and Wyatt, 1995). Bipolar patients showed reduced Na^+^/K^+^-ATPase *ATP1A2* gene expression in isolated erythrocytes (Hokin-Neaverson and Jefferson, 1989a,b; Looney and el-Mallakh, 1997) and in the temporal cortex (Rose et al., 1998) and reduced Na^+^/K^+^-ATPase *ATP1A3* expression in prefrontal cortex (Tochigi et al., 2008).

A prominent feature of manic episodes is hyperactivity. Although direct comparison of hyperactivity levels is impossible, all *Atp1a3* mouse models showed open field hyperlocomotion (Kirshenbaum et al., 2011a; Ikeda et al., 2013; Hunanyan et al., 2015).

Exploration-based anxiety tests revealed all *Atp1a3* mouse strains to be less anxious, to have increased impulsivity and risk-taking and a reduced habituation, all of which are symptoms of mania (Moseley et al., 2007; Kirshenbaum et al., 2011a; Ikeda et al., 2013; Hunanyan et al., 2015; Termsarasab et al., 2015).

In further support of mania-like behavior, the *Myshkin* mice showed changes to circadian rhythm (Kirshenbaum et al., 2011a). These symptoms, along with hyperactivity were reversed by treating the *Myshkin* mice with the mood stabilizers, lithium and valproate (Kirshenbaum et al., 2011a).

ICV injection of ouabain in rats caused phosphorylation of ERK and AKT in the hippocampus (Ruktanonchai et al., 1998; Kim et al., 2008; Yu et al., 2010). Similar increases in phosphorylated ERK and AKT were observed in the *Myshkin* mice. Both signaling pathways have been implicated in the control of behavioral excitement in rodents (Prickaerts et al., 2006; Creson et al., 2009; Engel et al., 2009; Ackermann et al., 2010), making them potential targets for future mood stabilizers. Correspondingly, open field hyperactivity and open arm visits were reduced after administration of the ERK inhibitor, SL327 (Kirshenbaum et al., 2011a). The *Atp1a3*-BAC transgenic *Myshkin* showed a Na^+^/K^+^-ATPase activity increase from 58 to 74% relative to wild type levels and a partial normalization of AKT phosphorylation (Clapcote et al., 2009; Kirshenbaum et al., 2011a). Correspondingly, treating the mice with the ouabain inhibitor, rostafuroxin, had a normalizing effect on hyperlocomotion (Kirshenbaum et al., 2011a).

Although the manic phase seems most prevalent in all mouse models, there have been examples of the *Atp1a3*^tm1Ling∕+^ mice being able to recapitulate the depression-like symptoms of bipolar disorder. When subjected to a chronic variable stress protocol, the mice showed prominent symptoms of anxiety, including reduced exploration of open areas and attention deficits during novel object and sociability tests. At this point, the mice showed a Na^+^/K^+^-ATPase activity of 67% relative to wild type levels (Kirshenbaum et al., 2011b). Interestingly, these symptoms did not occur in wild type mice, suggesting that *ATP1A3* mutations may increase vulnerability to stress.

## Epilepsy

Epilepsy and bipolar disorder share a common pathophysiology (Mazza et al., 2007) and is often comorbid in human patients (Mula et al., 2010). According to a recent study, approximately half of all AHC patients experience at least one epileptic seizure (Panagiotakaki et al., 2010).

Reduced Na^+^/K^+^-ATPase activity has been reported in genetic animal models of epilepsy and in hippocampal tissue from epileptic patients (Brines et al., 1995; Fernandes et al., 1996; Vaillend et al., 2002) and have been proposed as a causal factor in myoclonus epilepsy and ragged red fibers disease, a rare form of inherited epilepsy (McNamara, 1994). Also, Na^+^/K^+^-ATPase activity was decreased in the brain of rodents after chemical induction of seizures using the convulsant, pentylenetetrazol (Schneider Oliveira et al., 2004; Marquezan et al., 2013). Recently, two children with catastrophic early life epilepsy were shown to carry novel *ATP1A3* mutations (Paciorkowski et al., 2015).

As described, ouabain is convulsive when administered to rodents intraventricularly in sufficiently high concentrations (Doggett and Spencer, 1971; Davidson et al., 1978; Corazzi et al., 1985; Haglund and Schwartzkroin, 1990; Lees et al., 1990; Yu, 2003; Alonso et al., 2013).

Several mechanisms can explain why neurons are vulnerable to *ATP1A3* insults. Seizures are often associated with a loss in metabolic energy (Araujo et al., 2014). Na^+^/K^+^-ATPases are highly sensitive to such perturbations, as they require approximately 50% of the energy available to the brain under normal circumstances (Attwell and Laughlin, 2001). Reduced Na^+^/K^+^-ATPase activity may cause hyperexcitability due to increased [K^+^]_o_ and membrane depolarization (Haglund and Schwartzkroin, 1990; Somjen, 2002). Further effects arise from the post-tetanic buildup of [Na^+^]_i_ (Azarias et al., 2013) and the following inhibition of the Ca^2+^/Na^+^ exchanger causing accumulation of [Ca^2+^]_i_ and subsequent effects on gene transcription (Lyons and West, 2011), neurotransmitter release (Neher and Sakaba, 2008), and synaptic plasticity (Zucker, 1999).

The *Myshkin* mice showed epileptic seizures and neuronal hyperexcitability (Clapcote et al., 2009). Supporting the correlation between Na^+^/K^+^-ATPase α_3_ activity and seizure resistance, the epileptic seizures did not occur in *Atp1a3*-BAC transgenic *Myshkin* mice (Clapcote et al., 2009).

Furthermore, the link between *ATP1A3*-disease mutations and epilepsy was observed in a Chinese 12-year old boy with the I810N mutation, who was reported to have AHC with developmental delay and epilepsy (Yang et al., 2014).

The Mashl^+∕−^ mice showed flurothyl-indiced seizures, focal epileptogenesis (via kindling) and demonstrated spontaneous recurrent seizures and neuronal excitability (Hunanyan et al., 2015). Similar predisposition to epileptogenesis has been observed in humans affected by AHC.

There have been no reports of epilepsy in either of the *Atp1a3*^tm1Ling∕+^ and $\alpha_{3}^{+∕\Delta \text{E}2-6}$ mouse models (naïve or stressed), although the $\alpha_{3}^{+∕\Delta \text{E}2-6}$ mice showed increased sensitivity to cerebellar kainate injections (Ikeda et al., 2013).

Epilepsy is associated with cognitive decline in human patients (Bergen, 2006). Correspondingly, signs of hippocampal necrosis and glial activation were initially reported for the *Myshkin* mice maintained on the 129S1/SvImJ strain background. Hippocampal pathology disappeared once the mice were crossed into the C57BL/6NCr strain (Kirshenbaum et al., 2011a). Also, once maintained in the C57BL/6NCr strain for 20 generations, the *Myshkin* mouse strain no longer developed spontaneous seizures (Kirshenbaum et al., 2011a). This observation parallels previous publications identifying the 129S1/SvImJ and C57BL/6 strains as relatively resistant to kainate-induced seizures whereas only the C57BL/6 strain was resistant to kainate-induced cell death (Schauwecker, 2002; McLin and Steward, 2006).

## Memory

In a comprehensive report of 157 AHC patients, mental retardation was recorded in at least 92% of the cases (Panagiotakaki et al., 2010). Likewise, cognitive decline has been described in RDP patients (Cook et al., 2014).

Overall, naïve *Myshkin* and Mashl^+∕−^ mice displayed poor memory performance, whereas the performance of the *Atp1a3*^tm1Ling∕+^ and $\alpha_{3}^{+∕\Delta \text{E}2-6}$ mice to some degree was dependent on stress. The *Atp1a3*^tm1Ling∕^ mice thus showed no learning in locating a hidden platform in the Morris water maze (Moseley et al., 2007). In contrast the *Atp1a3*^*tm*1*Ling*∕+^ mice performed normally in a novel object recognition test, but showed significantly worse performance after subjection to a CVS protocol (Kirshenbaum et al., 2011b).

The *Myshkin* mice performed significantly worse in contextual- and cued-dependent fear conditioning tests. In contrast, Mashl^+∕−^ mice showed impaired novel object recognition, but intact cued-dependent fear memory (Hunanyan et al., 2015).

The dorsal part of the hippocampus plays a central role in learning and spatial memory, whereas the ventral hippocampus primarily regulates emotional and motivated behaviors through interaction with the amygdala (Fanselow and Dong, 2010). Accordingly, ouabain injection into these brain regions caused impairments in spatial learning (Zhan et al., 2004) and fear-dependent memory, respectively (Mizumori et al., 1987).

This could suggest a difference in the performance of the ventral hippocampus between the two *Myshkin* and Masl^+∕−^ strains. However, the strong hyperactivity of the both strains may have interfered with the readout of the conditioning tests, as the tests rely on the ability to suppress movement. Furthermore, the *Myshkin* mice showed reduced learning in a conditioned taste aversion test, suggesting also hippocampus-independent memory functions were affected (Reilly et al., 1993; Purves et al., 1995).

Due to a high voltage dependency and a high permeability for Ca^2+^, the N-methyl-D-aspartate receptors (NMDR) are important for triggering several different forms of synaptic plasticity, including long term potentiation and long-term depression (Cull-Candy et al., 2001). The *Atp1a3*^*tm*1*Ling*∕+^ mice showed reduced hippocampal expression of the NMDA NR1 subunit (Moseley et al., 2007), suggesting increased neuronal activity (Kvajo et al., 2004). Interestingly, NR1 expression was unaffected in the *Myshkin* mice (Clapcote et al., 2009). This observation is unexpected, as the *Myshkin* mice developed spontaneous seizures. However, direct comparison is not possible due to the FVB background of the *Myshkin* mice.

## Effects on neurotransmitter homeostasis and circuitry

As previously exemplified, decrease in Na^+^/K^+^-ATPase activity is associated with neuronal hyperexcitability and the release of neurotransmitters. Particularly dopamine, serotonin, and norepinephrine are involved in regulating movement and behavior (Perona et al., 2008).

In support of a dopamine phenotype, all naïve *Atp1a3* mouse models displayed hyperlocomotion in the open field test, which was further induced by amphetamine (Moseley et al., 2007; Kirshenbaum et al., 2011a). High Performance Liquid Chromatography (HPLC) analysis showed no changes in striatal levels of dopamine, serotonin, or their metabolites in the *Atp1a3*^*tm*1*Ling*∕+^ mice (DeAndrade et al., 2011). In cerebrospinal fluid (CSF) samples obtained from two RDP patients, low levels of the dopamine metabolite, homovanilic acid, was reported (Brashear et al., 1998). However, this observation remains to be confirmed as a diagnostic criterion for RDP. A recent study reported normal CSF neurotransmitter levels in AHC patients (Fons et al., 2012).

## Additional neurological symptoms

*Some ATP1A3*-disease mutation related patients show additional neurological symptoms that range from mild limb cramping sometimes decades before developing RDP (Brashear et al., 2007) to dysfunction of the autonomic nervous system with cardiac repolarization problems (Novy et al., 2014; Jaffer et al., 2015) excessive or lack of perspiration, skin discoloration, gastrointestinal problems and changes in body temperature leading up to or during attacks in AHC patients (Mikati et al., 2000; Fons et al., 2012).

The *Myshkin* mice showed increased systolic and diastolic blood pressure, but normal heart rate (Kirshenbaum et al., 2013). Most likely, this effect is related to *ATP1A3* expression in cardiomyocytes (Zahler et al., 1993). The Mashl^+∕−^ mice and stressed female *Atp1a3*^*tm*1*Ling*∕+^ mice showed delayed temperature response (DeAndrade et al., 2011; Hunanyan et al., 2015), suggesting impaired thermoception. The α_3_ Na^+^/K^+^-ATPase expression was recently reported in dorsal root ganglion γ motor neurons located in the spinal cord of mice (Edwards et al., 2013). It is therefore very likely to have implications for motor control also. Somatosensory evoked potentials (SEP) during the interictal period showed abnormal recovery cycle in a recent case report of seven AHC patients, suggesting multilevel somatosensory system hyperexcitability (Vollono et al., 2014). Sensory abnormalities have been proposed to play a role in the pathophysiology of dystonia as the basal ganglia and other motor areas are heavily connected to the somatosensory system (Tinazzi et al., 2003). Future studies may elucidate if a similar interaction affects the pathophysiology of *ATP1A3*-related diseases.

## Summary

The current *Atp1a3* mouse models recapitulate to a large part the symptoms of RDP and AHC.

Through the collaborative efforts of the *ATP1A3*-disease research community, it has recently been possible to carry out several studies on relatively large patient groups. Such studies continue to be invaluable not only in the search for common denominators but also for establishing the animal models for the ATP1A3-diseases.

Previous studies using CTS to study Na^+^/K^+^-ATPase function suggest a strong correlation between reduced Na^+^/K^+^-ATPase activity and severity of symptoms. The present *Atp1a3* mouse models seem to support this as the *Atp1a3*^*tm*1*Ling*∕+^ and $\alpha_{3}^{+∕\Delta \text{E}2-6}$ mice (with mild Na^+^/K^+^-ATPase reduction) showed relatively mild symptoms whereas the *Myshkin* and Mashl^+∕−^ models carrying AHC mutations (with larger Na^+^/K^+^-ATPase reduction), recapitulated most of the key phenotypes. As a proof of concept, most symptoms of the *Myshkin* mouse were rescued by increasing the Na^+^/K^+^-ATPase activity. Future experiments will be required to establish if similar approaches can be translated into a possible treatment.

Animal models represent valuable tools to study the pathology of human diseases. The biological questions can be assessed by different model system, depending on the nature of the study. Different advantages and disadvantages for designing and generating mouse models. Knock-out mouse models represent loss-of-function studies of a gene, while knock-in allows to explore the consequences of a single amino acid mutation inroduced into the genome. Both knock-out and knock-in models are can be designed as conditional models, allowing to knock-out, or introduce a mutation (knock-in) in single cell populations or organs, using the Cre LoxP system (Branda and Dymecki, 2004). Blocking Na^+^/K^+^-ATPase activity by infusion of ouabain (Fremont et al., 2014) or RNAi tools (Fremont et al., 2015) into specific brain areas represent a another model to investigate the function in specific brain areas in rodent models.

The highly variable nature of *ATP1A3*-disease related symptoms are becoming increasingly apparent. Despite recent advances in elucidating the etiology of individual *ATP1A3* mutations, large variations are reported even for patients with the same mutation. It is very likely that genetic background, epigenetic as well as environmental factors play a central role in disease penetrance.

## Author contributions

TH and KL discussed, outlined and co-wrote the review.

## Funding

Danish National Research Foundation (DNRF) (PUMPKIN DNRF85).

### Conflict of interest statement

The authors declare that the research was conducted in the absence of any commercial or financial relationships that could be construed as a potential conflict of interest.
